# Case report: Propylthiouracil-induced serious side effect: Perinuclear antineutrophil cytoplasmic antibody-associated vasculitis or IgA vasculitis?

**DOI:** 10.1097/MD.0000000000038790

**Published:** 2024-07-05

**Authors:** Wen Zhang, Xinyin Liu, Xiaoran Wang, Hongzhen Ma, Peipei Zhang

**Affiliations:** a Department of Nephrology, The First Affiliated Hospital of Zhejiang Chinese Medical University (Zhejiang Provincial Hospital of Chinese Medicine), Hangzhou, Zhejiang, China; b Department of Traditional Chinese Medicine, Jiande First People’s Hospital, Jiande, Hangzhou, Zhejiang, China; c Department of Nephrology, The First People’s Hospital of Hangzhou Lin’an District, Hangzhou, Zhejiang, China.

**Keywords:** antineutrophil cytoplasmic antibody-associated vasculitis, case report, Graves’ disease, IgA vasculitis, propylthiouracil, renal failure

## Abstract

**Introduction:**

Antineutrophil cytoplasmic antibody (ANCA)-associated vasculitis (AAV) is a rare disease characterized by the inflammation and destruction of small blood vessels and circulating ANCAs. Drugs such as antithyroid drugs (ATDs), especially propylthiouracil (PTU), have been used for the production of ANCAs and cause the development of drug-induced AAV. The pathogenesis of this disease is unclear but could be related to the physiological processes affecting the degradation of neutrophil extracellular traps (NETs). At present, PTU is widely used in patients with Graves’ disease (GD) who are preparing for pregnancy and whose condition has not been controlled. Once drug-induced AAV has occurred with important organ damage, considering NETs have a significant role in the immune system, whether the cessation of drugs could stop the progression of organ damage is unclear, and a consensus regarding standard treatment has not been established.

**Patient Concerns:**

In this case report, a female patient who planned pregnancy was hospitalized with multiple joint pain, impaired renal function, and hematuria. Immunofluorescence of the renal biopsy demonstrated spherical and diffuse mesangial distribution of IgA (3+). Autoimmune serology demonstrated positivity for autoantibodies against p-ANCA and an anti-MPO titer 74.72 RU/mL.

**Diagnosis:**

She was diagnosed with PTU-induced p-ANCA-associated and IgA-associated vasculitis (IgAV).

**Interventions:**

The patient accepted low doses of glucocorticoid, immunosuppressive therapy and RAI treatment.

**Outcomes:**

Both her kidney function and thyroid function remained were on the mend.

**Conclusion:**

The authors believe that this type of patient needs to fully consider their pregnancy preparation needs, suspend pregnancy when a small chance of GD remission is indicated, and avoid the use of drugs with reproductive toxicity and other serious adverse events. The multidisciplinary combination therapy of low-dose glucocorticoids and immunosuppressants combined with iodine radiotherapy is one reasonable scheme. At the same time, it is necessary to eliminate the organ damage caused by other reasons. This report provides a clinical treatment basis for patients with drug-induced vasculitis manifestations who cannot receive an accurate diagnosis.

## 1. Introduction

A female patient with Graves’disease (GD) who planned pregnancy was hospitalized for renal damage and multiple joint pain because of propylthiouracil (PTU). The diagnosis tended to be PTU-induced antineutrophil cytoplasmic antibody (ANCA)-associated vasculitis (AAV), which was charactered by necrotizing inflammation of small-to-medium blood vessels and possible granulomatous formation. AAV includes granulomatosis with polyangiitis (GPA), microscopic polyangiitis (MPA), and eosinophilic GPA. This disease pathogenesis could be associated with the physiological processes affecting neutrophil extracellular trap (NET) degradation.^[1]^ Secondary AAV is widely accepted to be typically mitigated upon withdrawal from this drug.^[2]^ However, some cases have reported that the disease progresses rapidly and is associated with unfavorable outcomes; plasma exchange and methylprednisolone are used to slow its progression.^[3]^ Notably, more than one case was presented as vasculitis in association with both ANCA positivity and immune complex deposition in renal tissue.^[4]^ Whether the disease can be defined as immune complex-related vasculitis deserves further discussion. Furthermore, the titer of MPO was not significantly high, and the renal biopsy of the patient demonstrated IgA distribution. Specific characteristics that allow detection of AAV in renal pathology were difficult to observe, preventing patients from receiving an accurate diagnosis and targeted treatments. PTU is currently widely used in patients with GD who are preparing for pregnancy but whose condition has not been under control. Once drug-induced AAV has caused substantial organ damage, it is unclear whether discontinuing drugs would stop the progression of organ damage, and no consensus on standard treatment has been reached. We provide an effective treatment plan that balances the patient pregnancy preparation needs and her condition for the first time, and discuss deeply how to prevent the occurrence.

## 2. Case presentation

A 28-year-old Chinese woman who is preparing for pregnancy presented to our hospital with multiple joint pain, impaired renal function, and hematuria. Her temperature was 37.5°C (on day 2, it was 37.3°C), her pulse was 72 beats per minute, blood pressure was 127/75 mm Hg, height was 158 cm, and weight was 55.4 kg. The patient had a history of GD. Her serum creatinine level at presentation was 130 μmol/L, urinalysis revealed 3 + occult blood and 2 + protein, the levels of thyroid function and ultrasound of the renal tract were not remarkable. The patient had progressive wrists, shoulders, interphalangeal joints, and ankle arthralgia, did not have any current symptoms of systemic illness, specifically significant fever, sensory disturbance, tinnitus, gross hematuria, dyspnea, cough, rash, skin purpura, Abdominal pain, melena, oral ulceration, or peripheral edema. The patient was transferred to the renal department for further investigation. Further inquiry revealed that her hyperthyroidism had been diagnosed 18 months prior without any relevant symptoms, and approximately 5 months prior, she was diagnosed with GD with high levels of thyroid-stimulating hormone-receptor antibodies (TSH-R-Ab) and thyroglobulin (Tg) at that time. She was initially treated with MMI and was changed to PTU 1 year before her pregnancy plan according to the 2018 European Thyroid Association (ETA) guidelines. Because of persistent low thyroid stimulating hormone (TSH) levels, and high TSH-R-Ab and Tg levels, the daily dose of PTU was escalated to 300 mg 1 month ago. She had no family history of vasculitis or nephritis.

**Figure 1.**
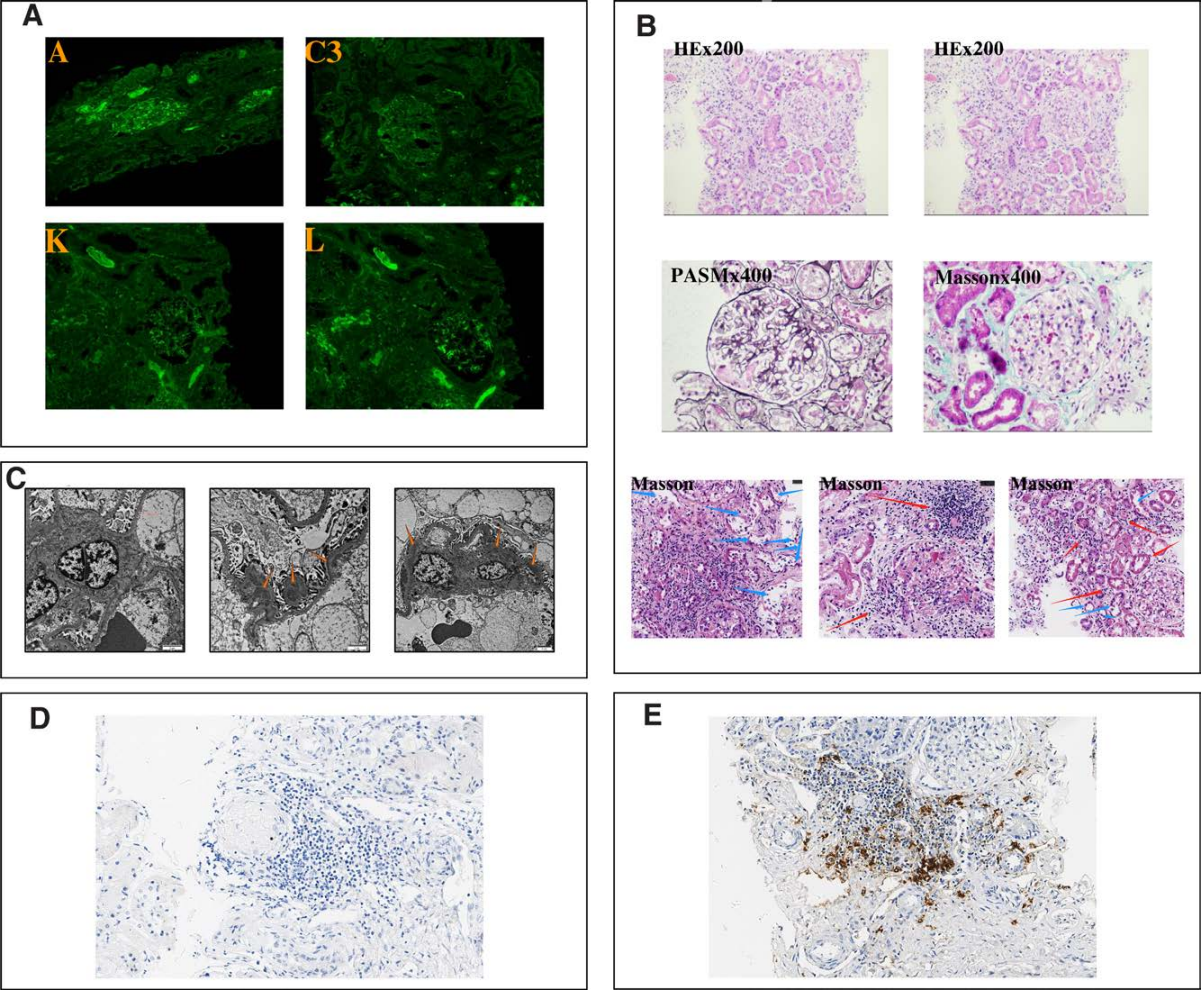
Kidney biopsy. (A) Immunofluorescence. (B) Light microscopy. (C) Electron microscopy. The arrows pointed to mesangial and subendothelial deposits. (D) Immunohistochemistry showed individual IgG and IgG4 positive plasma cells per high-power field. (E) Immunohistochemistry showed a large number of CD38-positive plasma cells per high-power field.

Immunofluorescence of the renal biopsy (Fig. 1A) demonstrated spherical and diffuse mesangial distribution of IgA (3+), mesangial positivity for C3 (1+), IgM (1+), lambda (3+), and kappa (2+). IgG, C1q, Fib, ALB, AA, C4c, IgG1-4, PLA2R, and THSD7A staining results were negative. Light microscopy (Fig. 1B) demonstrated cellular/fibrocellular/fibrous crescents in 5/24 glomeruli, with segmental necrosis and moderate chronic damage, widespread lymphoplasmacytic infiltrate associated with tubulitis, and electron microscopy (Fig. 1C) also demonstrated mesangial deposits and deposits in the basement membrane subendothelial of capillary loop. In view of the lymphoplasmacytic infiltrate, CD38, IgG, and IgG4 were subsequently performed by immunohistochemistry. This showed a large number of CD38-positive plasma cells and individual IgG and IgG4-positive plasma cells per high-power field (Fig. 1D–E).

Autoimmune serology demonstrated positivity for autoantibodies against p-ANCA and a high anti-MPO titer (74.72 RU/mL, reference range 0–20 RU/mL). According to the 2017 American College of Rheumatology/European Alliance of Associations for Rheumatology classification criteria for AAV (now validated for clinical research in 2022),^[5]^ we conducted systematic evaluation.

**Figure 2.**
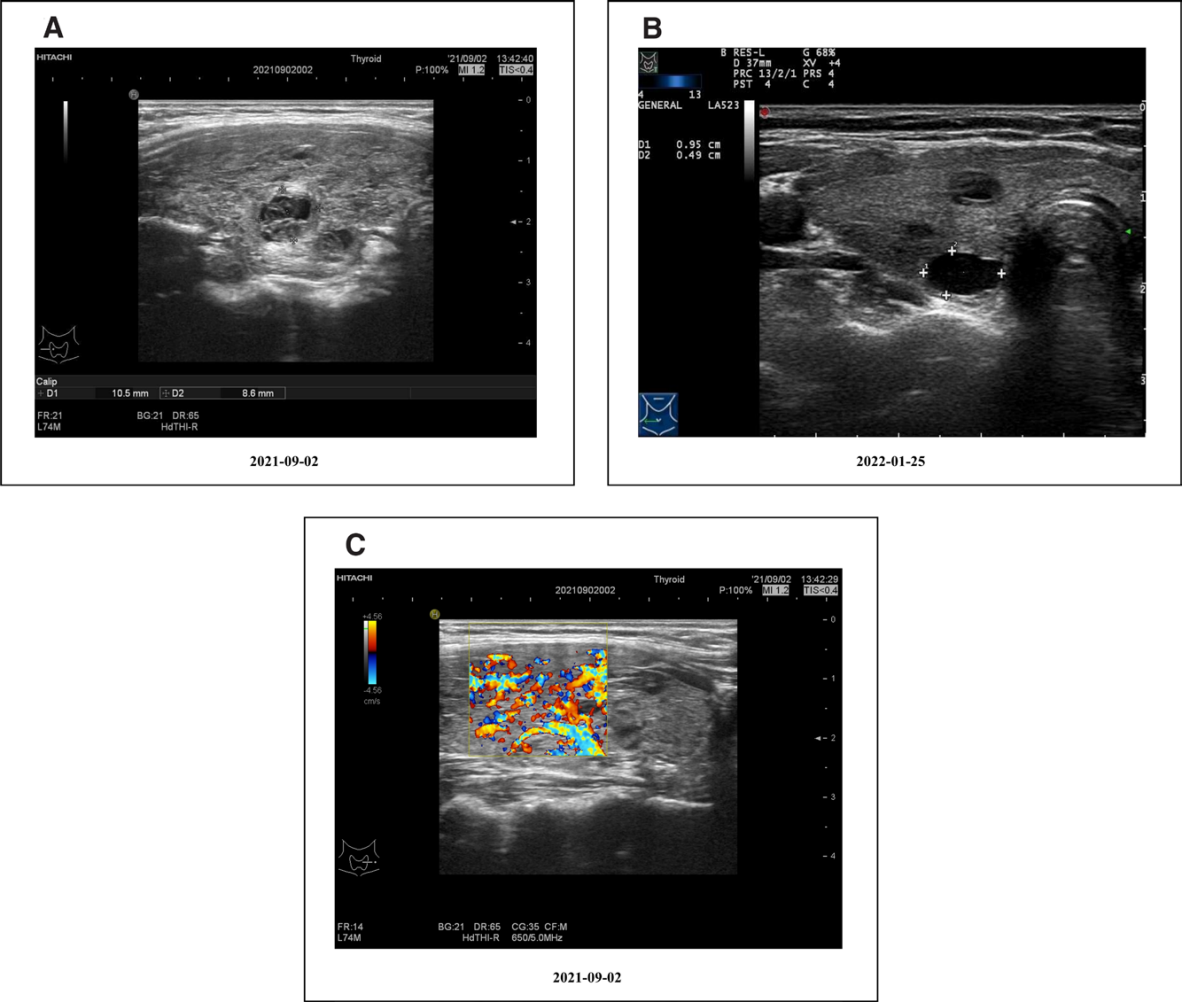
Thyroid ultrasound. (A) Ultrasonic examination of the thyroid showed diffuse thyroid lesions with volume enlargement. (B) Ultrasonic examination of the thyroid showed multiple cystic nodules of the thyroid. (C) Doppler examination of the thyroid showed the “thyroid inferno.”

No obvious abnormal changes were found on magnetic resonance imaging of paranasal sinuses. The external acoustic meatus examination was normal with normal hearing. The neurologic examination was negative. Chest imaging did not show pulmonary fibrosis or interstitial lung disease. Blood routine examination showed that eosinophil levels were 0.02 × 10^9^/L (<1 × 10^9^/L). Urinalysis repeatedly revealed occult hematuria and proteinuria. Fecal occult blood test was negative. These results pointed to the classification of MPA. Other laboratory tests showed the following: antinuclear antibody spectrum was negative, anti-cyclic citrullinated peptide antibody was0.5 U/mL, rheumatoid factor was < 20.00 IU/mL, C-reactive protein was10.1 mg/L, erythrocyte sedimentation rate was 18 mm/h, serum IgA was 2.58 g/L, serum IgM was 1.05 g/L, serum IgG was 13.10 g/L, complement C3 was 0.92 g/L, complement C4 was 0.41 g/L, serum creatinine was 126 μmol/L. Computer image generated [^99^mTc]diethylenethiaminepentaacetic acid estimated renal plasma flow (left kidney: 27.00 mL/min; right kidney: 35.03 mL/min), and [^131^I]orthoiodohippurate estimated renal plasma flow (left kidney: 120.59 mL/min; right kidney: 123.37 mL/min). Color-flow Doppler examination of the thyroid showed diffuse thyroid lesions with volume enlargement (Fig. 2A), and “thyroid inferno” (Fig. 2C).

### 2.1. Clinical course

The endocrinology and nephrology departments provided targeted multidisciplinary interventions. PTU was stopped, and methylprednisolone (MP) 40 mg intravenous drip per day for 7 days was initiated.

**Figure 3:**
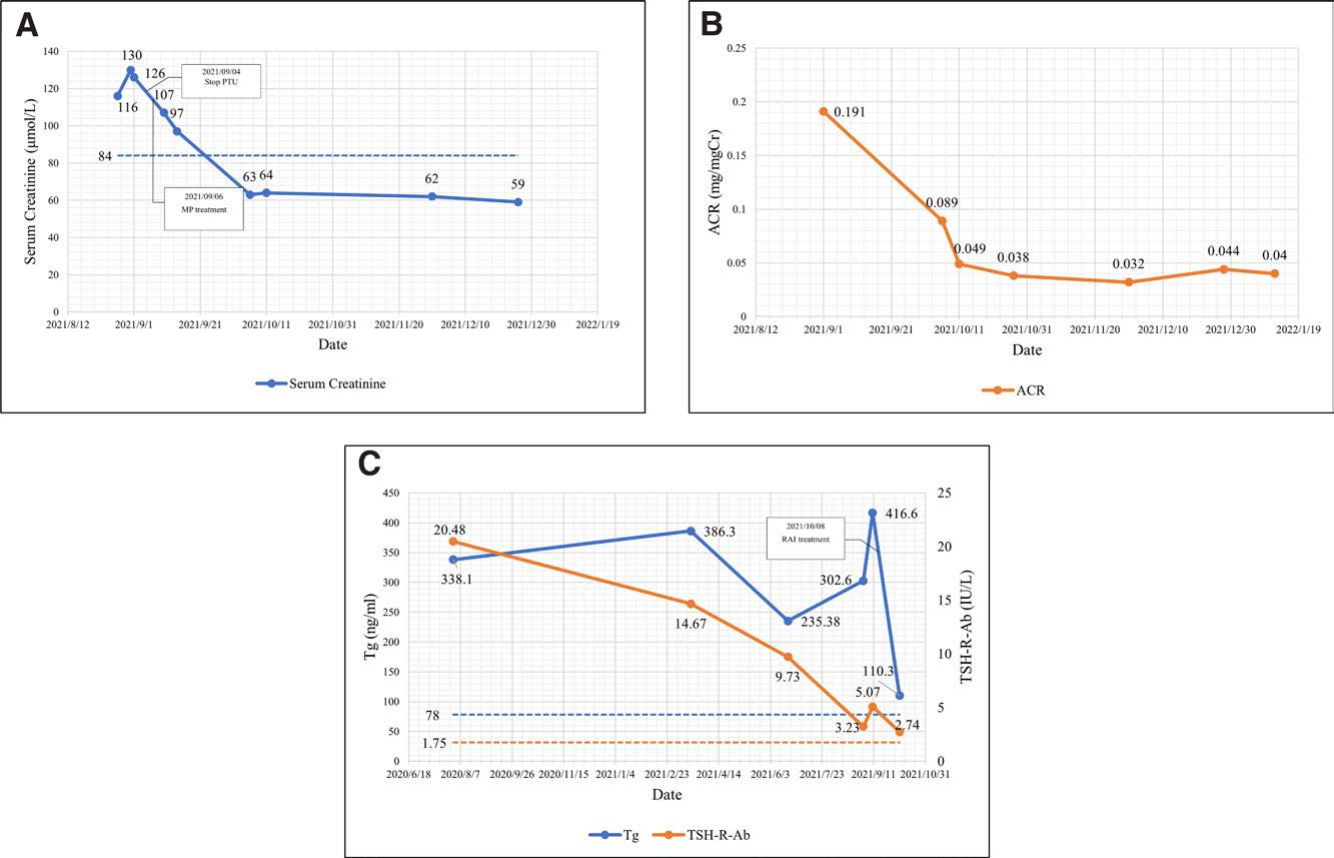
(A) Serum creatinine level. PTU, propylthiouracil; MP, methylprednisolone. Dotted lines mean upper normal limit of reference range. (B) ACR level. ACR, Urine Albumin-to-Creatinine Ratio. (C) Tg & TSH-R-Ab level. Tg, thyroglobulin; TSH-R-Ab, thyroid stimulating hormone-receptor antibodies. Dotted lines mean upper normal limit of reference range.

Her arthralgia got relief quickly, renal function continued to improve and stabilize with a serum creatinine level of 64 μmol/L, the titer for anti-MPO was 57.91 RU/mL, urinalysis revealed 3 + occult blood, negative protein and low urine albumin-to-creatinine ratio 1 month after the initiation of steroid treatment (Fig. 3B). The patient then provided consent to RAI treatment for GD.

## 3. Results

The patient was scheduled to follow up with nephrology and endocrinology outpatient clinics for maintenance of remission with a reducing course of oral prednisone and MMF. Her kidney function remained within the normal range (Fig. 3A) and thyroid function was on the mend. Tg and TSH-R-Ab also gradually declined (Fig. 3C), ultrasonic examination of the thyroid showed multiple cystic nodules of the thyroid without “thyroid inferno” (Fig. 2B). Then we conducted a follow-up on the patient for over 2 years, and her urine protein remained negative throughout.

## 4. Discussion

**Figure 4.**
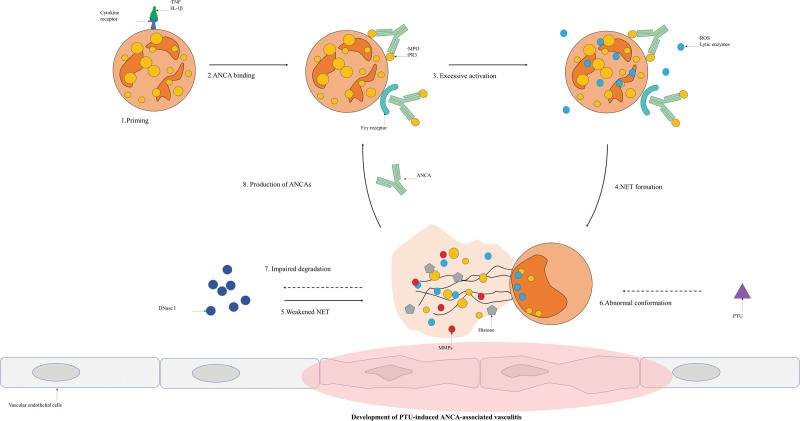
Development of PTU-induced ANCA-associated vasculitis. ANCA = antineutrophil cytoplasmic antibody, PTU = propylthiouracil.

The complications of PTU are diverse. Among the most common is secondary AAV, often characterized by joint pain and kidney damage. Drugs such as antithyroid drug (ATD), especially PTU, are involved in the production of ANCAs and cause the development of drug-induced AAV. The prevalence of ANCA-positive cases caused by ATD ranges from 4% to 64% in the PTU group (median, 30%).^[6]^ It is associated with the abnormal formation and impaired degradation of NETs. PTU usually induces the formation of abnormal degradation in NETs. The disordered regulation of NET is a major cause of ANCA production. In conclusion, the formation of NETs and ANCAs forms a vicious cycle in AAV pathogenesis (Fig. 4).^[1]^

This case presented with nonspecific clinical symptoms of joint pain and transient fever, and renal biopsy and laboratory results suggested the presence of overlapping diseases.

The elevation of p-ANCA, along with renal dysfunction and crescentic glomerulonephritis, suggested the possibility of secondary AAV. Joint pain, renal damage, and IgA deposition in the kidneys suggested the possibility of IgA-associated vasculitis (IgAV). Renal histopathology confirms a mesangiopathic process, immunofluorescent staining for IgA, which deposits on mesangial and subendothelial locations, along with crescents and endocapillary proliferation. Previous studies reported immune complex deposition in MPO-AAV. According to a study, 41% of cases had at least 1 Ig or complement component deposited in the glomeruli (immunostaining ≥ 2 + on a scale of 0 to 4).^[7]^ A study on immune complex deposits in AAV reported that deposits were limited to mesangial or mesangial plus subepithelial/intramembranous locations, and subendothelial deposits were rare based on electron microscopy.^[8]^ The patient had no purpura/petechia symptoms and abdominal pain; chest imaging was negative, and there was insufficient evidence to diagnose IgA vasculitis. As a result, the author is forced into a diagnosis and treatment conundrum.

Secondary AAV accompanying IgAV was rare in clinical practice. Ohta et al reported a case of a 16-year-old young woman who developed IgAV combined with AAV after 6 years of PTU use.^[9]^ Her renal biopsy findings were similar to those of the present case, showing crescentic glomerulonephritis and mesangial IgA deposition. Another young male patient developed serum positivity and purpura after PTU use, but IgAV was not confirmed by renal biopsy. Two additional young patients with GD developed ANCA positivity and exhibited purpura after PTU use, but IgAV was not confirmed through renal biopsy.^[10,11]^

It was unfortunate that we cannot determine whether the patient had subclinical IgAV before taking PTU. However, the patient condition improved remarkably. The urine protein turned negative in routine urinalysis after 1 month of treatment, which was rare in both acute primary IgAV and AAV. We leaned toward diagnosing this as a case of secondary p-ANCA and IgA-related vasculitis.

We finally decided on a multidisciplinary treatment strategy.

Endocrinology experts considered that, according to the 2018 ETA guidelines,^[12]^ the patient Tg and TSH-R-Ab levels are high and the color-flow Doppler examination of the thyroid showed “thyroid inferno,” suggesting that the probability of recurrence after stopping the drug is larger, which is not conducive to the patient condition. The ATD was discontinued, and iodine radiation therapy was switched to iodine radiation therapy.

Nephrology experts believe that stopping the drug is necessary, as the patient already has damaged kidney function; stopping the drug neither may not achieve a good treatment effect nor does it meet the patient desire to control the condition as soon as possible. Choose glucocorticoid to induce remission is appropriate since it is the classic method to induce IgAV and AAV remission. Chinese guidelines recommend AZA or MMF for maintenance of remission in MPO-ANCA-associated glomerulonephritis patients.^[13]^ A Study revealed the high 5-year patient and renal survival rates in Chinese MPA patients who received glucocorticoids plus MMF regimen for inducing and maintaining remission.^[14]^ After receiving the patient consent, we finally applied low-dose glucocorticoids combined with MMF to maintain remission.

Combination therapy was highly effective, the patient kidney function and thyroid function were within the normal range, Tg and TSH-R-Ab were gradually decreasing, and the “thyroid inferno” was becoming negative. During the follow-up of over 2 years, there were consistently negative results for urine protein.

## 5. Conclusions

This case report describes the clinical presentation, medical management, and outcomes of a female patient with PTU-induced p-ANCA and IgA-associated vasculitis who planned pregnancy. AAV occurred despite the fact that GD was treated in accordance with the 2018 ETA Guideline.^[12]^ It is unclear if the patient had an underlying original IgAN prior to the visit. The patient declined a repeat renal biopsy due to the need for pregnancy preparation and excellent treatment outcomes. We will follow up. Many questions remained unanswered in this case. We found that even if the renal biopsy was completed, it might not be able to make a clear diagnosis, resulting in a lack of standard treatment. The original treatment plan of patients with GD should, therefore, not be changed during pregnancy preparation. If necessary, the dose should be increased. Regular renal function tests, as well as routine blood and urine tests, are required. For patients with an unclear diagnosis, we recommend multidisciplinary treatment. Following renal function damage, the nephrology department should intervene in time, and at the moment, glucocorticoid-induced remission combined with subsequent immunosuppressive therapy may be the only clear and effective treatment. some latest guidelines recommend rituximab for primary AAV, but its effectiveness in secondary AAV is unknown. The radiology department needs to be involved if the thyroid gland is enlarged or its function is enhanced; RAI treatment is feasible.

## Acknowledgments

We would like to thank Editage (www.editage.com) for English language editing.

## Author contributions

**Writing – original draft:** Wen Zhang, Xinyin Liu

**Writing – review & editing:** Xiaoran Wang, Hongzhen Ma, Peipei Zhang
